# Healthcare Utilization in Older Adults: The Impact of Caregiving on Hospitalization Outcomes

**DOI:** 10.1093/geroni/igaf122.2830

**Published:** 2025-12-31

**Authors:** Erblin Shehu, Kanika Arora

**Affiliations:** State University of New York at Cortland, United States; University of Iowa, Iowa City, Iowa, United States

## Abstract

While current evidence suggests that caregiver involvement may reduce hospitalizations, few studies have examined differences in hospitalization outcomes based on caregiving arrangements or the causal directionality of this relationship. Using data from the Health and Retirement Study (HRS) (2006–2018; N = 22,792 observations), this study addresses this gap by conducting a two-part model to assess the longitudinal effects of caregiving on hospitalization risk and frequency among older adults with functional limitations. First, we apply a linear probability two-way fixed-effects model to evaluate whether different types of caregiver support (no help, non-family help, family help, or combined help) influence hospitalization likelihood. Then, among those hospitalized, a Poisson two-way fixed-effects model examines the impact of caregiver support on the number of hospital stays. Interaction terms explore variation by activities of daily living (ADLs), comorbidities, depression status, urbanicity, race, and sex, with all covariates lagged by one wave. While our findings indicate that caregiver type does not significantly impact hospitalization risk, we find that combined help reduces hospital stays by 17% among hospitalized individuals. Interaction effects reveal that older adults with high depression receiving combined help are 5% less likely to be hospitalized, whereas racial minorities receiving combined help experience a higher risk of hospitalization (Black: +4%, Other minority: +11%) and increased hospital stays (+20%). These findings suggest that integrated caregiver models can reduce hospital stays but may have differential effects based on mental health status and racial background.

